# Editorial: Pharmacological interaction between drugs and medicinal plants, Volume II

**DOI:** 10.3389/fphar.2024.1372366

**Published:** 2024-01-30

**Authors:** Maria Eva González-Trujano, Rosa Ventura-Martínez, Dâmaris Silveira, Myrna Déciga-Campos

**Affiliations:** ^1^ Laboratorio de Neurofarmacología de Productos Naturales, Dirección de Investigaciones en Neurociencias, Instituto Nacional de Psiquiatría Ramón de La Fuente Muñiz, Ciudad de México, Mexico; ^2^ Departamento de Farmacología, Facultad de Medicina, Universidad Nacional Autónoma de México, Ciudad de México, Mexico; ^3^ Laboratory of Natural Products, Faculty of Health Sciences, University of Brasilia, Brasilia, Brazil; ^4^ Sección de Estudios de Posgrado e Investigación, Escuela Superior de Medicina, Instituto Politécnico Nacional, Ciudad de México, Mexico

**Keywords:** herb-drug combinations, herbal medicine, pharmacokinetic interactions, pharmacodynamic interactions, synergism

## Introduction

**FIGURE 1:**
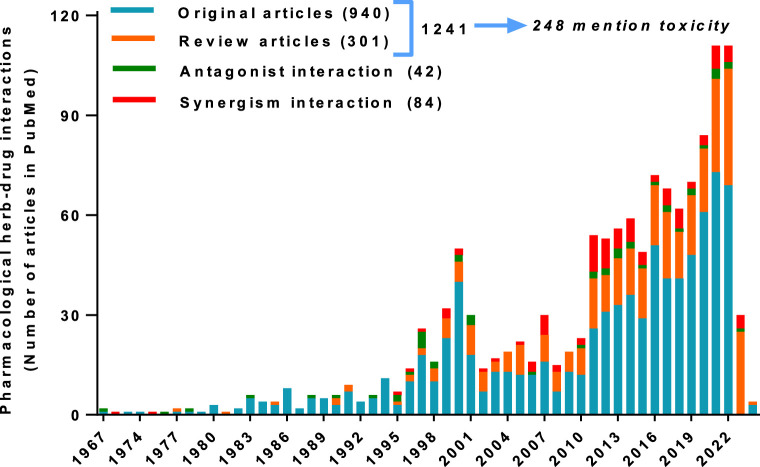
Number of reports of Pharmacological interactions herb-drug obtained from a search in the PubMed database.

In 2022, the herbal medicine market was valued at more than USD 148 billion and projected to reach around USD 165 billion in 2023 (Market Research Future, 2023). The countries with the greatest and most continuous traditional use of medicinal plants mainly include China, India, Brazil, and African countries. Regarding Traditional Chinese Medicine (TCM), the global revenue was almost USD 29 billion in 2022 projected to be USD 30.2 billion in 2023 (Persistence, 2023). Since the COVID-19 pandemic, the use of herbal medicine, already growing in the last decades due to the aging of the world population and the search for alternatives to conventional pharmacotherapy, has considerably increased. As pointed out in the prevoius editorial (Déciga-Campos et al., 2022), the demand for herbal medicines implies that more studies about plant-drug interactions must be conducted to improve efficacy and patient safety. Ever since the Research Topic “Pharmacological Interaction Between Drugs and Medicinal Plants Vol. I (2021–2022)” until now, little is found in literature reporting the subject “herb-drug interaction,” where most of the reports have been reviews. The present Research Topic named “Pharmacological Interaction Between Drugs and Medicinal Plants Vol. II (2022–2023)” contributes to the information about the herbal-drug interaction research with six articles that, directly or indirectly, show the importance of knowing about how the concomitant use of herbal medicine and drugs can impact -positively or not–on the pharmacotherapy of patients. As showed in Figure 1, it is integrated a total of 1,241 reports found using the PubMed Database from the period of 1967–2024 by considering the phrase “Pharmacological interaction of herb-drug”. These publications were original investigations (940) and reviews (301) with cases of antagonism (42) or synergism (84) in the pharmacological interactions, as well as 248 mentions of toxicity that emphasize the lacking and required pharmacovigilance of herbal therapy (Choudhury et al., 2023) alone or combined with other therapies.

Despite an apparently exponential increase observed in 2 decades (from 2002 to 2022), little information of this type has been reported in the last year (2023), where mainly reviews were published. This editorial shows evidence from six articles on this Research Topic as follows:

Zhao et al. reported an interesting systematic review, where authors present evidence that some Chinese Herbal Medicines (CHM) combined with antiepileptic drugs (AEDs) might improve the quality of life of patients presenting refractory epilepsy. The International League Against Epilepsy defines refractory epilepsy or drug-resistant epilepsy as a failure to respond adequately to two tolerated and appropriately chosen and used AEDs schedules. Although the pharmacological armamentarium has increased in AEDs classified as first, second, and third generation, many patients, mainly children, are considered to suffer drug-resistant epilepsy since it is not easily managed or relieved with the available drugs alone or combined. If a patient is still having seizures after taking more than two drugs, the possibility for adequate control of seizures is very low. Because of this, other treatment options should be considered; one of them might be a combination of herbal medicine and clinical drugs. CHM has been widely used as an adjunct to AEDs for intractable epilepsy. In the systematic review and meta-analysis of Zhao et al., the authors described the potential of these two therapies by analyzing randomized controlled trials by including twenty studies with 1,830 patients to describe monthly seizure frequency, abnormal rate of electroencephalogram, seizure duration, quality of life, and adverse effects. The authors integrated a table informing the composition of several preparations of the CHM, mentioning the plant species, the quantity of each, and the recommended dosage. Their results showed major efficient control of seizures without significant changes in duration but emphasizing less presence of adverse effects. However, the authors pointed out that most of the trials had poor methodological quality, requiring stronger evidence of this positive interaction of therapies by reports of studies of high quality to warrante better options for controlling drug-resistant epilepsy.

Not only clinical but preclinical studies of natural products combined with drugs have demonstrated that central nervous activity might involve neuroprotection and neuroplasticity processes. The original study reported by Vega-Rivera et al. described that neuroprotection is possible due to the ability of ellagitannins contained in the *Punica granatum* by activating β-estrogen receptors and because their antioxidant properties previously reported to prevent changes in the redox balance involved in the pathogenesis of depressive disorders (Cervantes-Anaya et al., 2022). In addition, these compounds also possess the ability to promote neural networks in the brain through growth and reorganization synaptogenesis. In the report of Vega-Rivera et al., the chronic administration of a combination of subtherapeutic doses of citalopram and a liofylized aqueous extract of *P. granatum* induced an antidepressant-like effect in ovariectomized female rats. At the end of the behavioral evaluation, the brains were dissected to analyze the formation and maturation of dendrite spines on granule cell dendrites and changes in the dendritic complexity. Their results showed not only the favorable antidepressant activity by using the combination of the natural product and drug but also an association with the increase in the dendritic complexity since the number of dendritic spines observed in the dentate gyrus of the hippocampus was enhanced, suggesting major synaptic connectivity that have been related to fast behavioral effects of antidepressant treatments. Furthermore, it was observed an increase in the mBDNF and synaptophysin expression, which has been reported as a biomarker of antidepressant efficacy, upregulated in structures of the limbic system such as the hippocampus and synaptogenesis induced by antidepressant drugs like citalopram, respectively.

Combination of therapeutic options might facilitate actions of natural products and clinical drugs not only at the central level. It has also been reported that several mechanisms of action might be activated to improve the therapeutic response in the peripheral nervous system. Ortiz reported the synergistic interaction of the α-bisabolol and diclofenac combination on the peripheral antinociception. In this study, the concomitant administration of an unsaturated monocyclic sesquiterpene alcohol as a natural product and a non-steroidal anti-inflammatory drug like diclofenac led to the potentiation of peripheral antinociception in rats by activation of different mechanisms of action that included the opioid receptors, nitric oxide, cyclic cGMP-K+ channels, and a biguanide-dependent mechanism. Pharmacological evidence has already reported that diclofenac activates the nitric oxide-cGMP antinociceptive pathway as well as its influence in the inhibition of the thromboxane-prostanoid receptor, affecting arachidonic acid release and uptake and inhibition of lipoxygenase enzymes, as well as the inhibition of substrate P, inhibition of peroxisome proliferator activated receptor gamma (PPAR-gamma), blockage of acid-sensing ion channels, alteration of interleukin-6 production, and inhibition of N-methyl-D-aspartate (NMDA) receptor (Gan, 2010). Whereas the α-bisabolol is one of the main bioactive components of the essential oil of flowers from species such as *Matricaria chamomilla*, a well-known medicinal plant and the most popular health-promoting herb for its nutraceutical components such as α-bisabolol, which pharmacological properties involve non-genomic and genomic mechanisms of action as a free radical scavenger and promoting their effects via reduction of TNF-α, IL-1β, IL-6, iNOS, and COX-2 and suppression of the activation of ERK1/2, JNK, NF-κB, and p38, among others (Ramazani et al., 2022). In the original research of Ortiz, a preclinical model of nociceptive pain in rats was used to determine the local peripheral antinociception by subcutaneous administration of α-bisabolol, diclofenac and their combination in the same paw as the nociceptive agent. The interaction between α-bisabolol and diclofenac was characterized by isobolographic analysis. The results showed that at the local peripheral level, the interaction between α-bisabolol and diclofenac produced a synergistic response in terms of antinociceptive action by involving different mechanisms of action.

It is important to mention that pharmacological combinations between medicinal plants, natural products, and clinical drugs are not specific for pharmacodynamic interactions. Pharmacokinetics also plays a relevant role in synergistic or even antagonistic responses for therapeutic purposes. The increase of clinical efficacy and reduction of toxicity of therapy are both objectives when treating complex diseases such as cancer. Chen et al. showed an original research suggesting that a Chinese herbal preparation named *Kangai* and irinotecan injection in colorectal cancer treatment might produce more efficacy when combined. *Kangai* is an herbal preparation integrated with oxymatrine, ginseng, and astragalus extracts. Irinotecan is a camptothecin derivative, whose interaction with the topoisomerase I-DNA complex induced cytotoxic effects. This preclinical study investigated the tumor volume of mice monitored daily to evaluate the therapeutic effect with the register of daily body weight, survival rate, hematopoietic toxicity, immune organ indices, and gut toxicity to identify the adverse effects by using a CT26 colorectal cancer-bearing BALB/c mouse model. Whereas, elucidation of the pharmacokinetic interaction of the herb-drug pair was analyzed in Sprague-Dawley healthy rats. Although co-administration with the Chinese herbal preparation conferred some protection against adverse effects caused by irinotecan, the results showed that Chinese herbal preparation did not protect irinotecan-treated mice from diarrhea and intestinal injury, and its efficacy remained limited. Thus, the authors declared that further studies on the pharmacokinetic behavior in other species should be performed to reproduce and reinforce their results.

The review article reported by Zhuang et al. included information related to the interaction of digoxin with several preparations used in Traditional Chinese Medicine (TCM). Digoxin is one of the most widely and commonly used drugs in treating heart failure and arrhythmia. It is one drug with a narrow therapeutic margin since its effective concentration in plasma is near the toxic concentration. Because of this, it is often used in combination with other drugs. However, drug interactions have promoted a significant impact on the plasma concentration of digoxin and may lead to the presence of adverse effects, such as poisoning. In this report, the authors analyzed 49 articles including clinical reports and pharmacological experiments using *in vitro* experiments. This manuscript describes details about the influence of TCM on the pharmacokinetic and pharmacodynamic properties of digoxin. Despite integrating referenced data, the authors found that these results are controversial due to the formulations used in the TCM. These medications have a complex composition and may interact differently with digoxin by increasing or diminishing its plasmatic concentrations that directly might affect the appearance of toxic effects by digoxin. This review article shows evidence about the interactions, both pharmacokinetics, and pharmacodynamics, between TCM formulations and digoxin to improve their efficacy and safety.

The last manuscript included in this Research Topic is an original research by Lei et al., where the authors showed the effects produced by a Chinese botanical drug preparation named Dahuang-Taoren (DT) containing two herbs: Rhubarb (dried rhizome of *Rheum palmatum* L.) and Peach kernel (dried mature seeds of *Prunus persica* L.) acting on the Rho GTPases in patients and in mice with adenomyosis (AM). Since the *in vitro* experiments showed the TCM downregulated RhoA, Rock1, CdC42, and Rac1, the *in vivo* confirmed the decreasing of Rho GTPase activity. AM is a condition that occurs when the cells that normally line the uterus grow into the muscular tissue of the uterine wall in reproductive-age women, promoting prolonged menstrual periods (menorrhagia), painful menstruation (dysmenorrhea), and chronic pelvic pain. This tissue growth has tumor-like features, where Rho GTPases can interfere with the destruction of tumor cells. The authors demonstrated that the DT granules induce a diminution in the expression of eRho GTPases in patients and mice, suggesting that this inhibition may be a potential option of treatment for the AM. Despite this study describing the interaction of herb-herb instead of an expected effect of an extract or a bioactive metabolite of an extract with a clinical drug, these results highlight the importance of using herbal preparations as a mixture of various medicinal plants and the complex pharmacological interactions that can be produced in benefit of specific pathologies.

With the advancing aging of the world population, new diseases, adverse effects, and the lack of effectiveness of many drugs, more herbal medicines will be used in combination with clinical drugs, among other therapeutic options. Efficient research to reinforce the efficacy and safety of combined treatment options such as herb-drug is a required practice for the scientific population to provide evidence of possible interactions, where an increase in financial support for this plant-drug interaction research is also imperative. Thus, it is essential to continue with the basic and clinical pharmacology research, as well as with the pharmacovigilance studies and the regulatory laws of this combined therapy.
